# COVID-19, A New Possible Mimicker of Interstitial Lung Disease Related to Primary Sjögren's Syndrome

**DOI:** 10.1155/2023/9915553

**Published:** 2023-12-09

**Authors:** Alessia Laneri, Stefania Cerri, Giovanni Della Casa, Antonio Moretti, Andreina Manfredi, Marco Sebastiani, Enrico Clini, Carlo Salvarani

**Affiliations:** ^1^Rheumatology Unit, University of Modena and Reggio Emilia, Azienda Ospedaliero-Universitaria Policlinico di Modena, Modena, Italy; ^2^Respiratory Diseases Unit and Center for Rare Lung Diseases, University of Modena and Reggio Emilia, Modena, Italy; ^3^Radiology Unit, University of Modena and Reggio Emilia, Modena, Italy; ^4^Rheumatology Unit, Irccs Arcispedale Santa Maria Nuova, Azienda Unità Sanitaria Locale-IRCCS di Reggio Emilia, Reggio Emilia, Italy

## Abstract

*Introduction*. Acute exacerbation of interstitial lung disease (ILD) and COVID-19 pneumonia show many similarities, but also COVID-19 sequelae, mainly when fibrotic features are present, can be difficult to distinguish from chronic ILD observed in connective tissue diseases. *Case Report*. In 2018, a 52-year-old woman, was diagnosed with primary Sjogren's syndrome (pSS). The patient did not show respiratory symptoms, and a chest X-ray was normal. During March 2020, the patient was hospitalized for acute respiratory failure related to COVID-19 pneumonia. Three months later, follow-up chest high-resolution computed tomography (HRCT) showed ground glass opacity (GGO) and interlobular interstitial thickening. Pulmonary function tests (PFTs) showed slight restrictive deficit and mild reduction in diffusion lung of carbon monoxide (DLCO). The patient complained of asthenia and exertional dyspnoea. A multidisciplinary discussion including rheumatologist, pulmonologist, and thoracic radiologist did not allow a definitive differential diagnosis between COVID-19 persisting abnormalities and a previous or new-onset pSS-ILD. A “wait and see” approach was decided, monitoring clinical conditions, PFTs, and chest HRCT over time. Only 2 years after the hospitalization, improvement of clinical symptoms was reported; PFT also improved, and HRCT showed almost complete resolution of GGO and interlobular interstitial thickening, confirming the diagnostic hypothesis of long-COVID lung manifestations. *Discussion*. In the above-reported case report, 3 differential diagnoses were possible: a COVID-19-related ILD, a preexisting pSS-ILD, or a new-onset pSS-ILD triggered by COVID-19. Regardless of the diagnosis, the persistence of clinical and PFT alterations, suggested a chronic disease but, surprisingly, clinical and radiologic manifestations disappeared 2 years later.

## 1. Introduction

Interstitial lung disease (ILD) is one of the most frequent and severe extraglandular complications of primary Sjögren's syndrome (pSS) [1]. pSS-related ILD shares many features with idiopathic forms of lung fibrosis, including the possible complication with an acute exacerbation (AE) [2]. Coronavirus disease (COVID-19) acute pneumonia is a severe complication of SARS-CoV-2 infection; it usually recovers completely but, in some cases, sequelae have been described, including persisting ground glass opacities or fibrotic changes. In these cases, differential diagnosis between long-COVID lung disease or a preexisting ILD may be difficult [3, 4].

Here, we describe a pSS patient who showed persisting features of ILD at high-resolution computed tomography (HRCT) after COVID-19.

## 2. Case Presentation

In 2018, a 52-year-old woman, with history of hypertension, was diagnosed with pSS. Her disease was characterized by dry eyes and mouth, positivity for anti-SSA (both 60 kD and 52 kD), anti-SSB, rheumatoid factor, and hypergammaglobulinemia, while the complement was normal. The pSS diagnosis was confirmed by a minor salivary gland biopsy, that showed a focus score 2, therefore the 2016 American College of Rheumatology/European League Against Rheumatism classification criteria for primary Sjogren's syndrome were also met [5]. The patient was treated with hydroxychloroquine (HCQ) until February 2020, then she stopped it for the appearance of drug-related myalgia.

**Figure 1:**
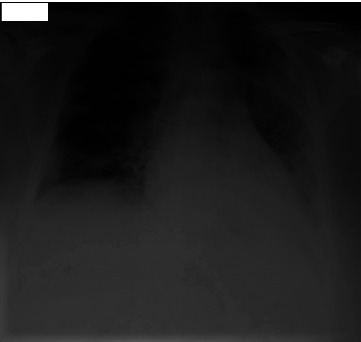
March 2020 chest X-ray. Large consolidation area at the left lung. Another consolidation area is visible at the right lung base. Large ground glass area affecting lower left lung, with minimal pleural effusion.

During March 2020, the patient was hospitalized for acute respiratory failure related to COVID-19 pneumonia (Figure 1). Her COVID-19 disease was classifiable as severe according to the COVID-19 treatment guidelines from the National Institute of Health [6]. Until then, the patient showed no respiratory symptoms and a previous chest X-ray was normal.

Three months later, in June 2020, a follow-up chest HRCT showed ground glass opacities and interlobular interstitial thickening. At the same time, pulmonary function tests (PFTs) showed mild alterations, with a restrictive deficit (FVC 78%) and a reduction in diffusion lung of carbon monoxide (DLCO 56%). The patient complained of asthenia and exertional dyspnoea. A multidisciplinary discussion including rheumatologist, pulmonologist, and thoracic radiologist did not allow a definitive differential diagnosis between COVID-19 persisting abnormalities and a previous undiagnosed or new-onset pSS-ILD. Therefore, a “wait and see” approach was decided, monitoring clinical conditions, PFTs, and chest HRCT over time. Treatment with HCQ was proposed again, while immunosuppressive treatment was delayed at that time.

Six months after acute onset of ILD, another HRCT showed stable findings. Eleven months later, clinical symptoms were unchanged, while PFTs and DLCO confirmed only mild alterations (FVC 81%, DLCO 56%).

**Figure 2:**
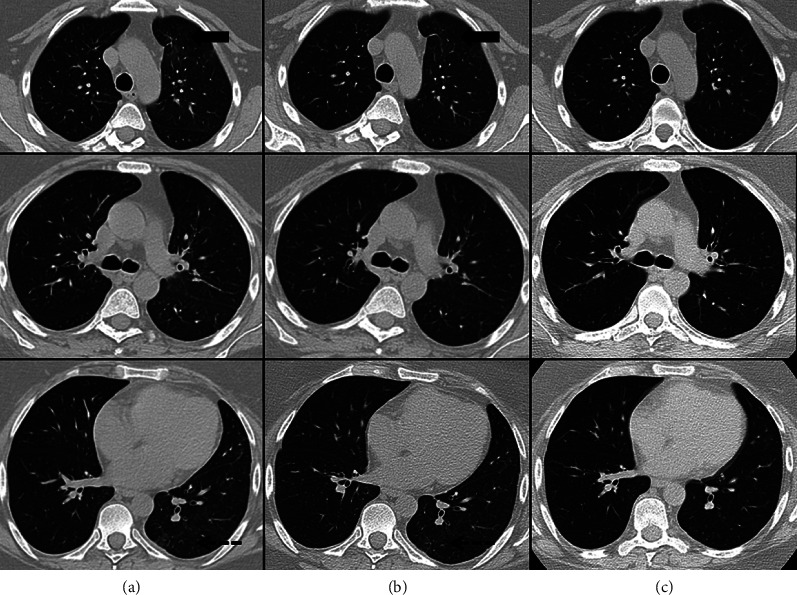
Three-(a), and six-month follow-up (b) chest HRCT images: stable ground glass opacity (GGO) and interlobular interstitial thickening, mainly in the peripheral-subpleural areas of the left lung; some fibrotic-disventilative stripes are unchanged at the anterior segment of the upper lobe (black arrow) and at the apical segment of the lower lobe, at the dorsal site (pointed arrow). Twenty-six-month follow-up (c) chest HRCT images: almost complete resolution of interstitial lung disease, and only a thin fibrotic-disventilative stripe remains at the anterior segment of the left upper lobe.

In March 2022, 2 years after the hospitalization for COVID-19, an improvement of clinical symptoms was reported; PFTs were also improved, FVC were normal, and DLCO significantly improved (see Table 1 for PFTs evolution); a new chest HRCT showed almost complete resolution of ground glass opacities and interlobular interstitial thickening disappeared, too (Figure 2).

Therefore, the clinical case was re-evaluated in a multidisciplinary discussion, confirming the diagnostic hypothesis of long-COVID lung manifestations.

## 3. Discussion

This case report adds new knowledge in the clinical history of SARS-CoV-2 lung involvement. First, COVID-19 sequelae may persist longer than previously observed; second, unlike other conditions, COVID-19 fibrotic lung disease can disappear also after a long time.

Several studies describe COVID-19 abnormalities persisting 1 year after the acute onset of the disease [7–11]. However, reports on both prevalence and features of post-COVID-19 lung fibrosis remain heterogeneous due to the different interpretation of CT abnormalities and to the heterogeneous definition of fibrotic changes [7].

A meta-analysis including 15 observational studies demonstrated that the proportion of residual CT abnormalities at 1-year follow-up was 37.7% in patients with a severe/critical COVID-19 disease and 20.7% in patients with a mild/moderate disease. In the second group, ground glass opacities were the major CT finding. In contrast, in patients with severe/critical COVID-19, fibrotic-like changes, bronchiectasis, and interlobular septal thickening were observed in addition to ground glass opacities. Considering the overall residual CT abnormalities at 1-year follow-up, in both groups, the most prevalent residual CT finding was GGO (21.2%), followed by fibrotic-like changes (20.6%). Interestingly, pulmonary function tests also showed a decline in DLCO as the major abnormal PFT finding 1 year after COVID-19, especially in severe/critical cases [8, 9].

In a recent multicentre, prospective, observational study evaluating DLCO impairment at 12 months from hospital discharge in COVID-19 patients, an improvement in DLCO values between 6- and 12-month follow-up was reported. A comparison between DLCO impairment and HRCT abnormalities was performed. It was observed that, among patients with DLCO impairment, 77% had HRCT abnormalities and, in particular, 61% of this patients group presented GGO while 49% presented reticular abnormalities on HRCT scans [11].

On the contrary, a recent multicentre retrospective study evaluating the global CT patterns, rather than individual findings of residual abnormalities in severe COVID-19 pneumonia survivors, showed that 45% of 405 patients had residual disease at 5–7 months after diagnosis, but only 7% had residual fibrotic abnormalities on follow-up CT scans. A follow-up longer than 11 months was available for 72 patients; among them, only 10, with fibrotic changes at 6-7 months, had unchanged CT scans at 12 months, while 1 patient showed a slight decrease in residual disease extension of fibrotic changes at 12-month follow-up CT scans [7].

Despite contrasting data, available published reports agree on the reversibility of the majority of COVID-19 residual CT abnormalities, even in the presence of fibrotic features [7, 10].

A recent multicentre prospective study assessed the persistence of symptoms up to 12 months after hospitalization for COVID-19 and showed that respiratory symptoms decline significantly over time. Respiratory symptoms, including dyspnoea, cough, and phlegm, were complained of by 87.3% of patients at 3 months; they declined to 79.1% at 6 months and to 76.0% at 12 months. Exertional dyspnoea was the most prevalent respiratory symptom reported by patients included in the study [12].

The presence of exertional dyspnoea was also evaluated by Faverio et al. However, in this study, an increase in the reported breathlessness was registered between 6- and 12-month visit [11]. A similar worsening trend was observed by another study that compared symptoms reported by patients with COVID-19 between 6 and 12 months after the disease onset, together with an increase in anxiety and depression. In contrast, an improvement of other symptoms, such as fatigue and muscle weakness, was reported (from 52% at 6 months to 20% at 12 months) [13].

In the above-reported case report, regardless of the diagnosis, the persistence of clinical and PFT alterations suggested a chronic disease but, surprisingly, clinical and radiologic manifestations completely disappeared after about 2 years. A “wait and see” approach was required to confirm the diagnosis together with a comprehensive multidisciplinary discussion.

In the diagnostic process, there were 3 possible differential diagnoses: a COVID-19-related ILD, a preexisting pSS-ILD or a new-onset pSS-ILD triggered by COVID-19.

Some similarities have been described between COVID-19 pneumonia and CTD-ILDs, including serological, clinical, radiological, and histopathological features. Radiological and histopathological findings of COVID-19 pneumonia are heterogeneous and include features resembling CTD-ILDs. [4]. The radiological characteristics of both are summarized in Table 2.

Pozzi et al. described three patients affected by CTD-ILDs whose respiratory failure was initially associated with SARS-CoV-2 infection. In these cases, there were no visceral manifestations except for lung damage and arthritis, so the correct diagnosis of autoimmune pneumonia, during the COVID-19 pandemic, was challenging. In all cases, the clinical conditions of the patients greatly improved after administration of immune-suppressive therapy. Considering the different management, a correct differential diagnosis between CTD-ILD and SARS-CoV-2 pneumonia is crucial to avoid a therapeutic delay and a subsequent potential worsening in prognosis [19].

A meta-analysis evaluating HRCT abnormalities from 2655 SARS-CoV-2 patients from 28 studies identifies GGO as the typical feature of COVID-19 (71.64%), possibly associated with consolidation, vascular enlargement, subpleural bands, interlobular septal thickening, pleural thickening, crazy paving pattern, bronchial wall thickening, and traction bronchiectasis. Typically, COVID-19 lung abnormalities are bilateral and peripheric, with an apical-basal gradient of distribution [14]. When COVID-19 pneumonia progress to acute respiratory distress syndrome, patchy confluent areas of GGO and consolidations with a typical anteroposterior gradient appear on HRCT scans [15].

CTD-ILDs present lung abnormalities comparable with COVID-19 pneumonia; similar radiological findings described in COVID-19 pneumonia are detected in some forms of CTD-ILDs [20]. In pSS-ILD, HRCT could show a variable combination of GGO, consolidations, reticular abnormalities, honeycombing, cysts, nodules, and bronchiectasis [1]. In particular, a recent retrospective study analysing interstitial lung involvement in 66 pSS patients, reported GGO on HRCT scans in 87.88% of patients; irregularities in pleural margins were present in 21% of patients, septal/subpleural lines in 81.82%, honeycombing in 28.79%, and subpleural cysts in 40.90% [16].

The most frequent radiological pattern, in patient with pSS-ILD, is nonspecific interstitial pneumonia (NSIP) [1, 16]. Fibrosing NSIP, the most frequent radiological pattern in CTD-ILDs, is characterized by a bilateral, symmetric distribution, with lower lung-zone predominant reticular opacities and traction bronchiectasis [17].

When AE-ILD occurs, chest HRCT scans show new extensive bilateral GGOs, sometimes accompanied with consolidations, with a variable distribution (peripheral, multifocal, or diffuse) [18], mimicking ARDS in COVID-19 pneumonia.

Therefore, in some cases, differential diagnosis from pSS-ILD and COVID-19 might represent a challenge for the physicians.

Immune-mediated mechanisms may influence both clinical course and long-term sequelae of SARS-CoV-2 infection, and the appearance of autoantibodies might predict adverse clinical course in these subjects [4].

In conclusion, only long-term observational studies could clarify the hypothesis that SARS-CoV-2 infection can act like a trigger for ILD in predisposed subjects, for example in rheumatoid arthritis or CTDs.

## Figures and Tables

**Table 1:** Percentage of predicted FVC and DLCO at months 4, 11, 20, and 26 after the acute onset of disease. FVC and DLCO values were stable for the first 11 months, then they slowly improved.

Months	4 (%)	11 (%)	20 (%)	26 (%)
FVC	78	81	85	89
DLCO	56	56	73	72

**Table 2:** Typical HRCT finding, lesions distribution, and acute exacerbation features on HRCT scans in COVID-19 and pSS-ILD from various studies [14–18].

	COVID-19	pSS–ILD
Typical findings		
GGO	71.64%	87.88%
Vascular enlargement	65.41%	—
Subpleural bands	52.54%	—
Interlobular septal thickening	43.28%	—
Septal/subpleural lines	—	81.82%
Pleural thickening/irregularities in pleural margins	38.25%	21%
Consolidation	29.15%	—
Crazy paving pattern	28.74%	—
Subpleural cysts	—	40.90%
Honeycombing	—	28.79%
Bronchial wall thickening	20.71%	—
Traction bronchiectasis	20.36%	—
Parenchymal micronodules/nodules	14.84%	92.42%
Distribution	Bilateral, peripheral distribution	Bilateral, symmetric distribution
Acute respiratory distress syndrome/acute exacerbation	Patchy confluent areas of GGO and consolidations with a typical anteroposterior gradient	New extensive bilateral GGOs, sometimes accompanied with consolidations, with a variable distribution (peripheral, multifocal or diffuse)

— = no available data.

## Data Availability

The data used to support the findings of the study are available from the corresponding author upon request through email.
